# Physical and Tensile Properties of Handmade *Sida rhombifolia* Paper

**DOI:** 10.1155/2020/3967641

**Published:** 2020-07-14

**Authors:** P. W. Huisken Mejouyo, E. Dydimus Nkemaja, O. R. Beching, N. R. Sikame Tagne, T. Kana'a, E. Njeugna

**Affiliations:** ^1^Laboratory of Mechanics (LM), ENSET-University of Douala, Douala, Cameroon; ^2^Laboratory of Mechanics and Appropriate Materials (LAMMA), University of Douala, Douala, Cameroon; ^3^Industrial Systems and Environmental Engineering Laboratory (UR-ISIE), IUT/FV Bandjoun-University of Dschang, Dschang, Cameroon

## Abstract

This study focuses on the production and characterization of biodegradable handmade paper from the *Sida rhombifolia* plant (SRP) cellulose. *Sida rhombifolia* plant is a seasonal plant that grows in the equatorial and tropical climates. The studies carried out on this SRP were aimed at investigating the methods required for the production of handmade paper from SRP plant and also at determining the tensile strength. Four specimens of SRP paper of different additive labels S0 (no additive), S1 (starch and KOH), S2 (starch), and S3 (Foska liquid glue) were produced using the Kraft method. Tensile properties (stress at break, elongation at break, and Young's modulus), the rate of water absorption, and the rate of moisture absorption were carried out. Results showed that the addition of potassium hydroxide considerably reduces Young's modulus of SRP handmade paper (S1) while the Foska liquid glue (S3) significantly improves it. In addition, the addition of potassium hydroxide and Foska liquid substantially improves the water absorption properties of the paper S2 and S3, respectively. The adhesive liquid creates more porosity and consequently increases the absorption of water. The addition of potassium hydroxide and Foska liquid significantly embedded the rate of moisture absorption. From the results obtained, it can be concluded that the paper S3 can be used as packaging paper since it has better mechanical properties and moisture absorption.

## 1. Introduction

According to some research studies on the suitability of aquatic plant fibres for handmade papermaking [1], in many countries, quantities of available wood are insufficient to meet the requirements and demands of pulp and paper especially in Mediterranean countries like Spain, Italy, and Greece. In Malaysia, over one million tons of papers were produced in 2005 according to Roda and Rathi [2]. This would mean that more tropical trees need to be felled to sustain papermaking industry to meet the paper requirement and demand. The felling down of more tropical trees causes deforestation and loss of rainforest. To reduce the loss of rainforests, an attempt was made to find the alternative source of fibre for paper. Some alternatives have been used to replace the wood fibre with non-wood-derived fibres according to Enayati et al. [3], from agriculture residues such as wheat and rice straw, sorghum stalks, jute, and hemp for paper production. Another alternative source of fibres for paper production is from aquatic plants found in lakes, ditches, rivers, ponds, and estuaries. They have short life cycle, grow massively, and due to their abundance can cause problems in irrigation channels and water bodies according to the authors in [4, 5].

Handmade paper industry is an environmentally friendly and very promising industry for local entrepreneurship. Still in the alternative to replace wood fibre for paper production, *Sida rhombifolia* plant (SRP) cellulose will be used as nonwood plant for papermaking. This plant is available in almost of all the ten regions of Cameroon. Many authors present *Sida rhombifolia* as a plant with extraordinary medical properties [6–8], notably extracts from stems, leaves, and roots. Unfortunately, in the absence of a local pharmaceutical industry exploiting these extracts, it was impossible for us to work with waste. So, we worked with crushed stems. Hence, the objectives of this study are to produce paper from SRP and characterize this paper. The derived values from the test shall follow TAPPI [9] and ISO standards. More so, the handmade paper produced from selected *Sida rhombifolia* plant (SRP) will be subjected to some mechanical (tensile strength) and physicochemical (water and moisture absorption) tests for quality control. Studies have been done on the characterization of fibre from the stem of *Sida rhombifolia* [10] and this paper is focused on the production of paper from the entire plant excluding the leaves and roots.

## 2. Materials and Method

### 2.1. *Sida rhombifolia* Plant

**Figure 1:**
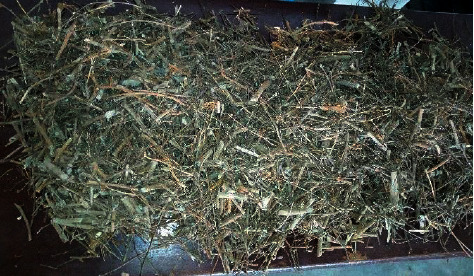
Chop dried stems (SRP).

The material used for the pulping is SRP harvested in Bamenda, a town in the northwest region of Cameroon. The harvested plant had stems and branches of about 30 mm of diameters. Leaves were removed from the stems and branches and then chopped into lengths of about 40 mm and dried for two weeks (Figure 1). The dried stems were ground into fine chips.

The chemical composition of the *Sida rhombifolia* plant (SRP) fibre (Table 1) is cellulose, hemicellulose, and lignin, which plays the role of adhesive. Cellulose and hemicellulose are the principal elements constituting pulp and paper.

### 2.2. Method for Pulp Production

The method of pulp and paper production that has drawn our attention was the Kraft method [11]. This method allows us, according to the literature review, to obtain a high strength paper. The Kraft method (sulfate process) is a transformation of wood into wood pulp, which has pure cellulose as a principal element. The Kraft method uses wood chips with a hot mixture of water, sodium hydroxide, and sodium sulfate (Table 2) that breaks the bonds which link hemicellulose, lignin, and cellulose. According to McDougall et al. [12], the Kraft method produces papers with increased fibre strength and density and low electrical conductivity [11].

### 2.3. SRP Handmade Paper Production

**Figure 2:**
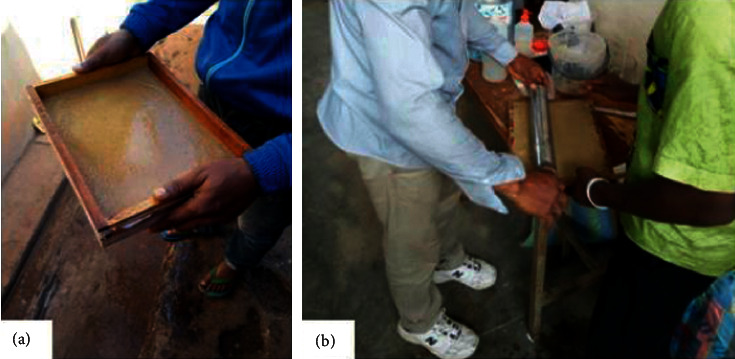
Paper production methods: (a) pulp in soluble state poured in the mould and (b) laminating pulp in the mould.

The production of SRP paper was done in two ways:

- The pulp was mixed with some additives in two litres of fresh distilled water and poured in a mould of 317 × 230 × 3 mm and then agitated for about 10 to 15 min for evenly distribution of the pulp on the mould and then about 65% of water was drained (Figure 2(a)). And after, it is dried in an oven at 45°C for 72 hours and then removed to obtain paper
- About 80% of water was removed by squeezing the pulp to have a paste, and then the paste is mixed with some additives and spread in a mould of 317 × 230 × 3 mm to be laminated with a roller to obtain a uniform thickness all through (Figure 2(b)) and then dried for 72 hours at 45°C in an oven.

Table 3 summarizes some volumes and masses of additives used in the production of paper.

### 2.4. Determination of Grammage, Density, and Microscopic Observation of the SRP Handmade Paper

Grammage is defined as the weight in grams per unit area of paper or board expressed as g/m^2^. Grammage and thickness are significant properties in the sale and use of the paper product.

According to ISO 186 [9], grammage (g/m^2^) of a paper or paperboard can be calculated as follows:

(1) $$\begin{array}{c} g=\frac{m}{A}\times 10^{6}, \end{array}$$

where *m* is the mass of the specimen in grams and *A* is the average area of the specimen in square millimetres. Density according to ISO 186 is determined using the following equation [9]:

(2) $$\begin{array}{c} \rho =\frac{m}{V}\times 10^{9}, \end{array}$$

where *m* is the mass of the specimen in grams and *V* is the average volume of the specimen. We also observed the microscopic structure of SRP handmade paper using the Celestron LCD Digital Microscope with 200x magnification.

### 2.5. Tensile Test of the SRP Handmade Paper

The sample test strips were sized according to ISO 1924-1, ISO 1924-2, and Technical Association of the Pulp and Paper Industry (TAPPI T 404) standards for tensile testing of paper and paperboard [13].

The samples were designed using the following dimensions:

- Jaw holding length up and down (*b*)=20 mm
- Test length (*L*)=100 mm
- Width (*w*)=15 mm

A tensile test was carried out using a specific tensile test machine for natural fibres and paper made in our laboratory [14]. It enabled to make the static tensile testing of fibres or paper samples whose length is between 10 and 250 mm. It is equipped with a load cell and allowed a gradual loading in steps of 0.25 N to a maximum capacity of 25 N. The four specimens produced, namely, S0, S1, S2, and S3 each had 10 test samples which were mounted in the upper mobile and down fixed jaws of the tensile test equipment. The application of load *F* is done until the rupture occurs.

The test values of the four specimen labels S0, S1, S2, and S3 allow us to plot a strain-stress diagram of each sample.

### 2.6. Water Absorption Test of the SRP Handmade Paper

**Figure 3:**
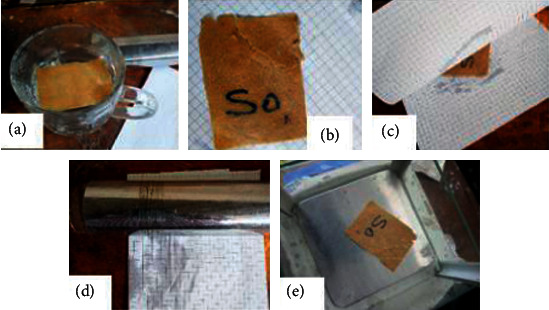
Water absorption test: (a) immerse sample, (b) sample placed on a dry sheet, (c) sample in between dry sheets of paper, (d) removing saturated water on the sample, and (e) weighing of the sample.

The water absorption test was made by immersion according to T 491 concerning the water immersion number of paperboard [15]. This test method is used for unsized paper (less or greater than 25 × 20 mm = 500 mm^2^) and paperboard having relatively high water sorption (noncoated paper). Here, the paper samples were weighed on an electronic balance for initial mass to be recorded and then were immersed in distilled water of 300 ml, and after 1 min, the sample was removed and placed in between two sheets of paper on a horizontal flat surface and then a roller of mass 500 g is rolled over once to remove the water on the two surfaces of the immerse sample (Figure 3). Then, the sample was weighed again and the mass was recorded. The exercise continues until the weight is stable.

The values obtained were for 10 samples of each specimen made. These values were recorded and the mass ratio was calculated and plotted against time to observe the sorption behaviour of the samples.

The values that shall be calculated here are as follows [16]:

(3) $$\begin{array}{c} \text{MR}=\frac{M_{t}-M_{i}}{M_{f}-M_{i}},\\ \text{WR}=\;\frac{M_{f}\;–\;M_{i}}{M_{i}}\times 100, \end{array}$$

where MR is the mass ratio; *M*_t_ is the mass at time *t*; *M*_*f*_ is the final stable mass during the test; *M*_*i*_ is the initial mass before the test; WR is the water absorption rate.

### 2.7. Moisture Absorption Test on the SPR Handmade Paper

The moisture absorption test was done here with respect to TAPPI standard T 402 with the relative humidity (RH) of 74.0 ± 2.0%.

Our samples were preconditioned in an oven at 23°C for 24 hours and then removed and put in a saturated sodium chloride (NaCl) container isolated from RH greater than 50.0%. These samples' weight shall then be measured on an electronic balance every hour until the weight becomes stable. The results obtained will be tabulated and a log time graph was plotted to show the increase of weight.

These values were recorded and a mass ratio was calculated and plotted against time to observe the moisture absorption behaviour of the specimens.

The values that shall be calculated here are as follows:

(4) $$\begin{array}{c} \text{MR}=\frac{M_{t}-M_{0}}{{\text{MS}}_{f}-M_{0}},\\ \text{HR}=\;\frac{M_{f}\;–\;M_{i}}{M_{i}}\times 100, \end{array}$$

where HR is the humidity absorption rate.

## 3. Result and Discussion

### 3.1. Pulp and Paper Obtained

Kraft method was used to obtain paper pulp. The two shaping methods allowed us to obtain different papers (S0, S1, S2, and S3) by incorporating the different additives as shown in Table 3.

**Figure 4:**
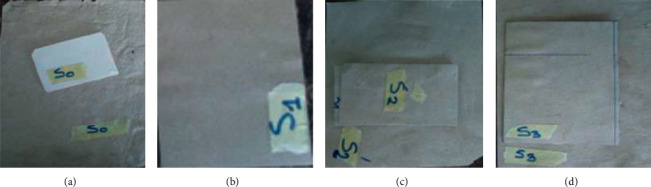
Samples of the paper obtained.

The results of the paper obtained are presented in Figure 4.

### 3.2. Physical Properties of Our Samples

**Figure 5:**
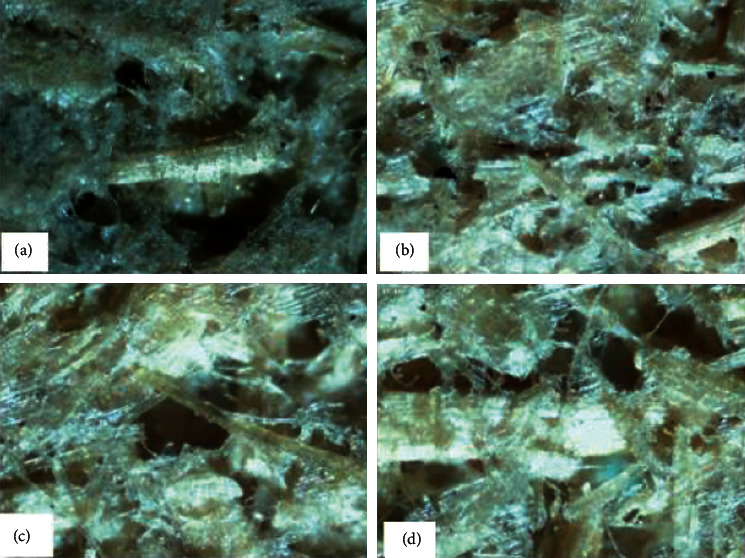
Microscopic observation of SRP handmade paper: (a) S0, (b) S1, (c) S2, and (d) S3.

Microscopic observations made using Celestron LCD Digital Microscope with 200x magnification are shown in Figure 5.  Table 4 presents the thickness, density, and grammage of SRP handmade paper.

Microscopic observations highlight a random fibrous structure. The presence of numerous micropores in the structure of the paper was noticed. Structurally, it seems to have no significant difference from one type of paper to another.

From Table 4, according to ISO standards for paper and paperboard, packaging paper thickness is above 0.30 mm and the grammage is above 200 g/m^2^; therefore, all our specimens' papers could be used for packaging.

### 3.3. Tensile Properties of SRP Handmade Paper

**Figure 6:**
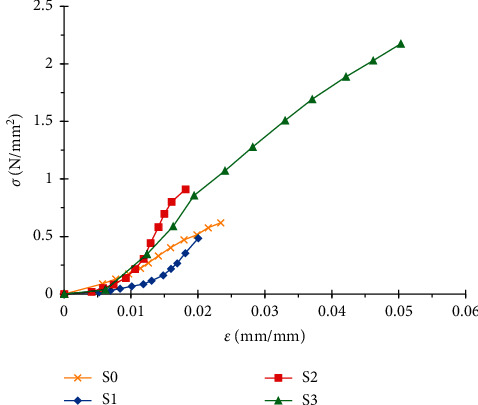
Summary graph of the 4 SRP handmade paper specimens.

The tensile test curves for the different papers are shown in Figure 6.

**Figure 7:**
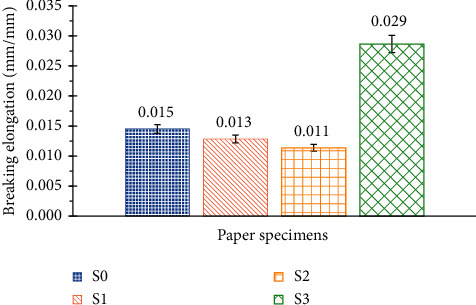
Breaking elongation of the 4 SRP handmade paper specimens.

**Figure 8:**
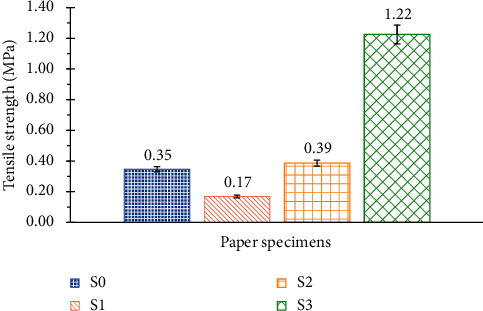
Tensile strength of the 4 SRP handmade paper specimens.

**Figure 9:**
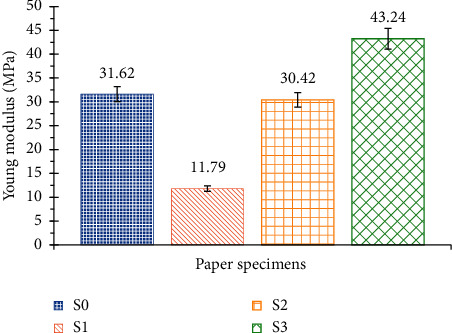
Young's modulus of the 4 SRP handmade Paper specimens.

These curves highlight the brittle behaviour for all paper specimens. The different mechanical characteristics obtained from the tensile test, notably the elongation at break, the stress at break, and Young's modulus, are summarized in Figures 7–9.

It is noticed in Figure 7 that starch and potassium hydroxide do not have a significant effect on the elongation at break (S0, S1, and S2). Furthermore, the addition of the liquid adhesive Foska improves significantly (approximately twice) the elongation at break (S3).

It can be seen in Figure 8 that the potassium hydroxide negatively affects the tensile strength (S1) while the Foska liquid glue significantly (substantially by four) improves it (S3). Starch does not have a significant effect on the tensile strength (S2).

It is noticed in Figure 9 that the addition of potassium hydroxide considerably reduces Young's modulus of SRP handmade paper (S1) while the Foska liquid glue (S3) significantly improves it (around 38%). Furthermore, the addition of 2% starch (S2) has no effect on Young's modulus. Moreover, the Kraft method retains only cellulose. However, recent studies [18, 19] proved that hemicellulose acts as a binding agent and increases the strength of the paper. This might be one of the reasons that the lack of binding agent (S0) showed poor results compared with the paper with adhesive (S3). Foska liquid glue certainly acts as a binder between cellulose microfibrils and improves internal cohesion. This may explain that the mechanical performance of S3 paper is better. Starch also plays the role of the binder but with weak cohesion forces. It is easy to understand why S3 is more resistant than papers containing starch (S1 and S2). Potassium hydroxide would have a weakening effect on cellulose microfibers and therefore generate a poor level of resistance.

### 3.4. Result and Discussion for Water Absorption Test Done

**Figure 10:**
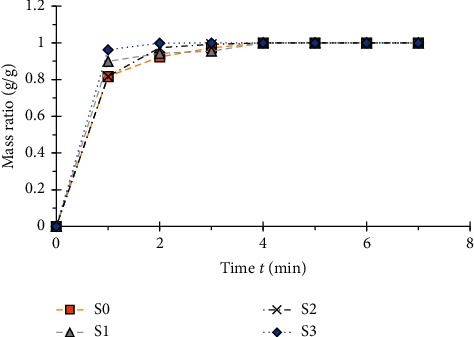
Summary graph of water absorption mass ratio of the specimens.

The different curves of the water absorption ratio are presented in Figure 10.

**Figure 11:**
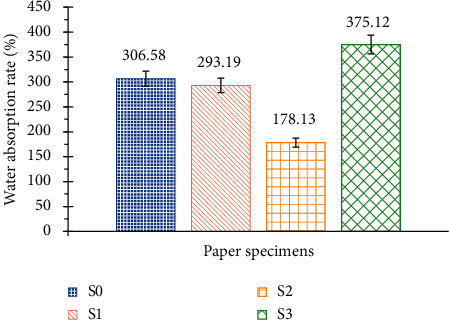
Water absorption rates of the 4 specimens.

It can be seen in Figure 10 that all the paper samples have two phases of water absorption. During the first minute, the absorption is very fast. Then the absorption becomes slow and all the samples reach saturation around the fourth minute.  Figure 11 shows different water absorption rates.

Figure 11 shows that the addition of starch and potassium hydroxide (S2) substantially improves the water absorption properties of the paper while the addition of starch alone (S1) has no noticeable effect. On the other hand, the addition of Foska liquid glue (S3) causes a great absorption of water. The liquid adhesive is certainly degraded by water, which creates more porosity and consequently increases the absorption of water.

### 3.5. Result and Discussion for Humidity Test Done

**Figure 12:**
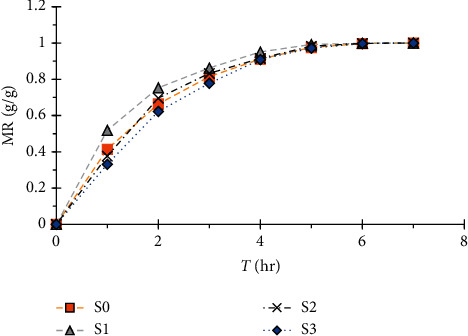
Moisture absorption curves of the 4 specimens.

The different curves of the humidity absorption ratio are presented in Figure 12.

Figure 12 shows that moisture absorption is slower than the absorption of water although it is always made in two phases: the absorption phase proper, which takes place during the first four hours, and then the stabilization phase, which ends around the seventh hour.

**Figure 13:**
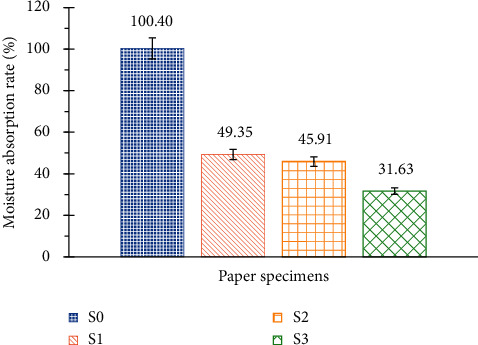
Bar chart of moisture absorbency of the specimens.

Figure 13 shows that the addition of starch and/or potassium hydroxide as well as Foska liquid glue improves the resistance to humidity. As the absorption of moisture takes place more slowly, there is no degradation of the liquid adhesive, and consequently, the humidity has little effect on the paper sample S3.

## 4. Conclusion

This research work investigates the possibility of manually made paper from the stems of *Sida rhombifolia*. Some physical (grammage and density) properties of this paper allow us to position it with regard to the ISO standard (ISO 536) applicable in this field as packaging paper. The mechanical properties permit us to define the resistance class of this paper although it can be improved. In addition, the sorption properties of the paper have been measured and allow us to conclude that by reducing the thickness, we can easily use it as a coffee filter since it does not disintegrate on contact with water. This paper and in particular that of class S3 can be used as packaging paper since it has better mechanical properties and lower moisture absorption compared to the other paper samples. Other tests may be carried out in future work to better assess the properties and the use of this paper.

## Figures and Tables

**Table 1:** Chemical composition of *Sida rhombifolia* fibre [10].

Cellulose (%)	Hemicellulose (%)	Lignin (%)	Wax (%)	Moisture content (%)	Density (kg/m^3^)	Ash (%)
75.09	15.43	7.48	0.49	12.02	1320	4.07

**Table 2:** Materials used in the pulping (Kraft process).

Raw material	Process	Chemical used	Conditions
Dried fine chips of SRP (100 g)	Open cooking (2 hours) On electric cooker	20% NaOH 18% Na_2_S 1500 ml of HO_2_	Wash in 10 L of freshwater and blending

**Table 3:** Additive mixed with SRP pulp for paper production.

Specimens (S)	2% starch	4% KOH	Foska liquid glue (300 ml)
S0	No	No	No
S1	Yes	Yes	No
S2	Yes	No	No
S3	No	No	Yes

**Table 4:** Physical properties of our specimens compared to ISO standards.

Specimen properties				ISO standards (paper and paperboard for packaging) [17]	
Specimen	Thickness (mm)	Density (g/cm^3^)	Grammage (g/m^2^)	Thickness (mm)	Grammage (g/m^2^)
S0	0.92	0.22	206.5	>0.30 mm	>200 g/m^2^
S1	1.03	0.30	310. 2		
S2	1.01	0.36	361.1		
S3	1.45	0.31	447.7		

## Data Availability

The data used to support the findings of this study are available from the corresponding author upon request.
